# Realization of Z_2_ Topological Photonic Insulators Made from Multilayer Transition Metal Dichalcogenides

**DOI:** 10.1021/acsnano.4c09295

**Published:** 2024-11-18

**Authors:** Tommi Isoniemi, Paul Bouteyre, Xuerong Hu, Fedor Benimetskiy, Yue Wang, Maurice S. Skolnick, Dmitry N. Krizhanovskii, Alexander I. Tartakovskii

**Affiliations:** † Department of Physics and Astronomy, University of Sheffield, Sheffield S3 7RH, U.K.; ‡ School of Physics, Engineering and Technology, University of York, York YO10 5DD, U.K.

**Keywords:** photonic crystals, tungsten disulfide, metasurfaces, topology, 2D materials, FDTD

## Abstract

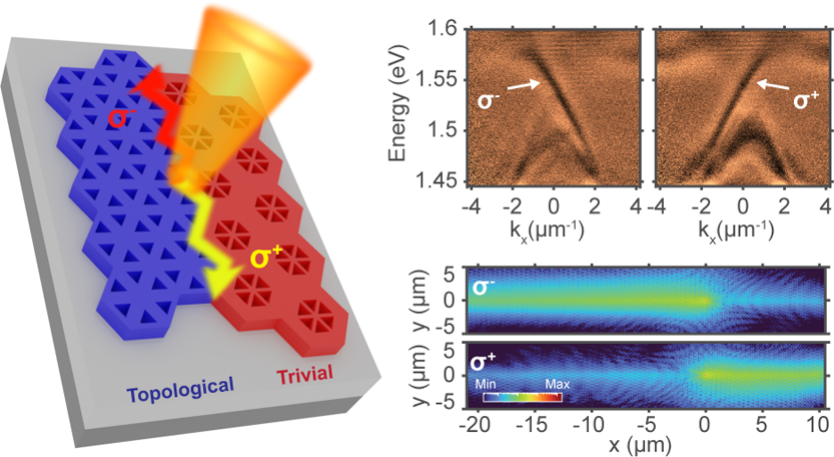

Monolayers of semiconducting transition metal dichalcogenides (TMDs) have long attracted interest for their intriguing optical and electronic properties. Recently, TMDs in their quasi-bulk form have started to show considerable promise for nanophotonics thanks to their high refractive indices, large optical anisotropy, wide transparency windows reaching to the visible, and robust room temperature excitons promising for nonlinear optics. Adherence of TMD layers to any substrate via van der Waals forces is a further key enabler for the nanofabrication of complex photonic structures requiring heterointegration. Here, we use the attractive properties of TMDs and realize topological spin-Hall photonic lattices made of arrays of triangular nanoholes in 50 to 100 nm thick WS_2_ flakes exfoliated on SiO_2_/Si substrates. High-quality structures are achieved by taking advantage of anisotropic dry etching dictated by the crystal axes of WS_2_. Reflectance measurements at room temperature show a photonic gap opening in the near-infrared in trivial and topological phases. Unidirectional propagation along the domain interface is demonstrated in real space via circularly polarized laser excitation in samples with both zigzag and armchair domain boundaries. Finite-difference time-domain simulations are used to interpret optical spectroscopy results. Our work demonstrates the feasibility of more complex nanophotonic devices based on the layered (van der Waals) materials platform.

## Introduction

Over the last two decades, layered crystals, often referred to as van der Waals materials, have attracted tremendous interest due to their favorable properties in mono- and few-layer forms. In particular, semiconducting transition metal dichalcogenides (TMDs) exhibit robust excitons with high oscillator strengths as well as direct bandgaps in monolayers making them attractive for integration in various photonic structures.^1^ Examples of such integration of TMD mono- and bilayers include realization of exciton-, trion-, and dipolar polaritons in dielectric microcavities,^2−5^ lasing in III–V semiconductor nanocavities,^6^ single photon emitters in monolayers coupled to III–V semiconductor nanoantennas,^7^ and polaritons in spin-Hall topological photonic crystals made from silicon-on-insulator^8^ and suspended silicon nitride^9^ structures.

The quasi-bulk counterparts of 2D materials have been much less explored but have recently started attracting considerable attention for their favorable optical properties with a potential for photonic applications (see e.g., refs (10−13)). Similar to monolayers, van der Waals layers of any thickness from few atomic layers up to 100s of nanometers and lateral sizes up to few 100 μm can be readily fabricated via mechanical exfoliation.^10−12^ Thanks to van der Waals forces, the exfoliated flakes can easily adhere to a wide range of substrates without the need for chemical bonding or lattice matching.^10−12,14^ By now, there are many demonstrations of standard electron-beam lithography followed by wet or dry etching used to pattern 2D materials leading to high-quality structures.^10−12,15,16^ Furthermore, such patterning can take advantage of etching anisotropy to produce crystallographically exact edges.^12,15,17^

Compared to Si or III–V semiconductors, semiconducting TMDs exhibit higher refractive indices,^12,18^ enabling confinement of light to smaller volumes; far larger birefringence values,^12,18^ attractive for light polarization control and nonlinear optics; transparency in the visible and near-infrared;^12,18^ out-of-plane van der Waals adhesive forces which offer additional postfabrication tuning^17^ and unconventional approaches to structure fabrication such as vertical layer and structure stacking and twisting^12^ similar to few-atomic-layer thick van der Waals heterostructures,^19^ which may enable the realization of previously inaccessible photonic structures. Finally, strong room temperature excitonic transitions in most semiconducting van der Waals materials^12,18^ open their potential for nonlinear nanophotonic elements.

Having demonstrated the range of favorable material properties offered by van der Waals crystals, in this work, we introduce these materials in the realm of topological photonics. This field emerged following the ideas that were first developed to understand topological phases of matter in the solid state physics starting with the discovery of the integer quantum Hall effect and then, 20 years later, of topological insulators.^20^ The evolution of the concepts initially applied to electrons led to the engineering of artificial magnetic fields (gauge fields) acting on photons and created using specially designed modulated photonic lattices.^21−28^ Topological photonic devices introduce additional functionality in nonlinear and quantum photonic applications, thanks to the unidirectional photonic edge states at their interfaces, which have inherently low scattering losses and backscattering-immune propagation, allowing, for example, scattering-free transport of light around tight bends and the possibility to form chiral light–matter interfaces.^21−28^ Photonic analogs of the spin-Hall^29^ and valley-Hall^30^ effects have been proposed. In experiments, such topological photonic interfaces have been demonstrated in photonic crystal structures fabricated in standard silicon-on-insulator wafers^26,27^ and suspended GaAs membranes.^23,28^

Here, we have designed and fabricated topological spin-Hall lattices in quasi-bulk WS_2_ exfoliated straight on a SiO_2_/Si substrate. Our approach resembles the robustness of the silicon-on-insulator platform,^26,27^ with the crucial advantage of a wide range of thicknesses of WS_2_ crystals available in a single exfoliation step, allowing an additional degree of freedom, the layer thickness, in designing topological structures for a given target wavelength. The use of a high-refractive-index van der Waals material also allows us to avoid the complication of fabrication of nanophotonic structures in ultrathin-suspended membranes necessary for GaAs.^23,28^

We have directly observed topological photonic states in the near-infrared spectral range in WS_2_ spin-Hall lattices with armchair- and zigzag-shaped domain walls. Topological interface modes are observed both in angle-dependent reflectivity measurements and resonant real-space propagation. Unidirectional propagation lengths as large as 9 μm are recorded, in spite of the use of inherently leaky spin-Hall structures in this initial demonstration. This propagation length is comparable with recently reported lengths in structures of a similar design made on a silicon-on-insulator substrate (11 μm),^31^ while from the data reported for suspended SiN membranes, propagation lengths of up to few tens of μm can be estimated.^32^ We carry out finite-difference time-domain (FDTD) simulations to reproduce and interpret optical spectroscopy results, describing both the photon bandstructure and real-space propagation.

Our work introduces complex nanophotonic devices in the van der Waals materials platform. The specific features of WS_2_ that we rely on for the presented demonstrations are (i) the high refractive index (>4) allowing good confinement of the modes and their relatively weak dependence on the surrounding material thus avoiding the need for suspended structures; (ii) low absorption in the near-infrared important for large propagation lengths; (iii) straightforward adhesion to SiO_2_ via van der Waals forces ensuring ease of fabrication; (iv) the ability to produce flakes of various thicknesses allowing large tunability of the photonic modes via the photonic crystal slab thickness, which can be used as an optimization parameter. Among this combination of advantages for photonics, we find that point (iv) specifically proves to be an important technical advantage at the research stage (requiring fast turnaround of studied samples) compared to silicon-on-insulator and III–V materials, where the slab thickness cannot be easily varied.

## Results and Discussion

**Figure 1:**
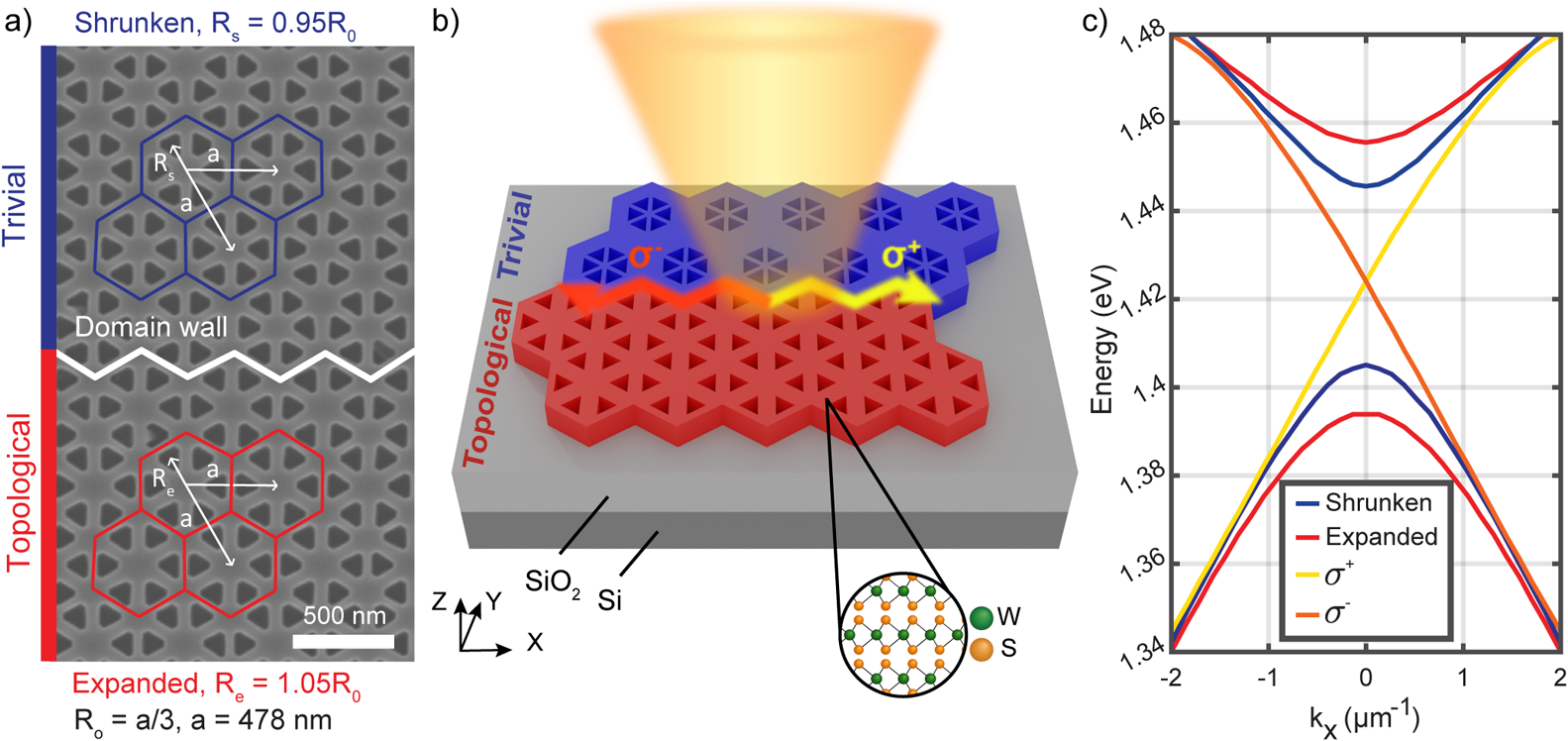
(a) Schematic of the hole lattices showing the trivial and topological domains and domain wall overlaid on a scanning electron micrograph of patterned WS_2_ flake with a thickness of 71 nm on SiO_2_/Si. (b) Schematic diagram of the structure and optical excitation at the interface. (c) Photonic band structure of the device in (a) at *k*_*y*_ = 0 in different domains and at the interface where two modes polarized in σ^+^ and σ^–^ are observed (*x* is the direction of the interface).

The considered photonic structures, presented in Figure 1a,b, consist of WS_2_ flakes used as slab waveguides confined by total internal reflection, patterned as topological lattices. The topological spin-Hall lattice consists of assemblies of 6 triangle-shaped holes patterned on the flake,^8^ which when arranged in a symmetric orientation, lead to a photonic band structure with a gapless Dirac cone at the Γ point. In the shrunken configuration, the lattice is perturbed by moving the triangles inward at each unit cell, which opens the gap with modes of a trivial topological nature. In the expanded configuration, the triangles are moved outward, opening the gap with inverted bands compared to the shrunken configuration, leading to nontrivial topology (Figure 1c). At the interface of the two topologically distinct regions, two states appear within the gap of the band structure, which allows propagating modes.^23,33^ Due to the spin-Hall effect, the interface states are accessible for either the counterclockwise (σ^–^) or clockwise (σ^+^) polarizations, allowing the control of the propagation direction (Figure 1b). The considered spin-Hall structure is leaky since the states are above the light cone at Γ point. This allows direct probing of optical modes using reflectivity measurements, including propagation in real space.^32^ Topological interface states can also be realized in valley-Hall structures, where the states are at the K point below the light cone. This allows for better confinement, but separate coupler structures would be required for the detection of the edge states.^34^

**Figure 2:**
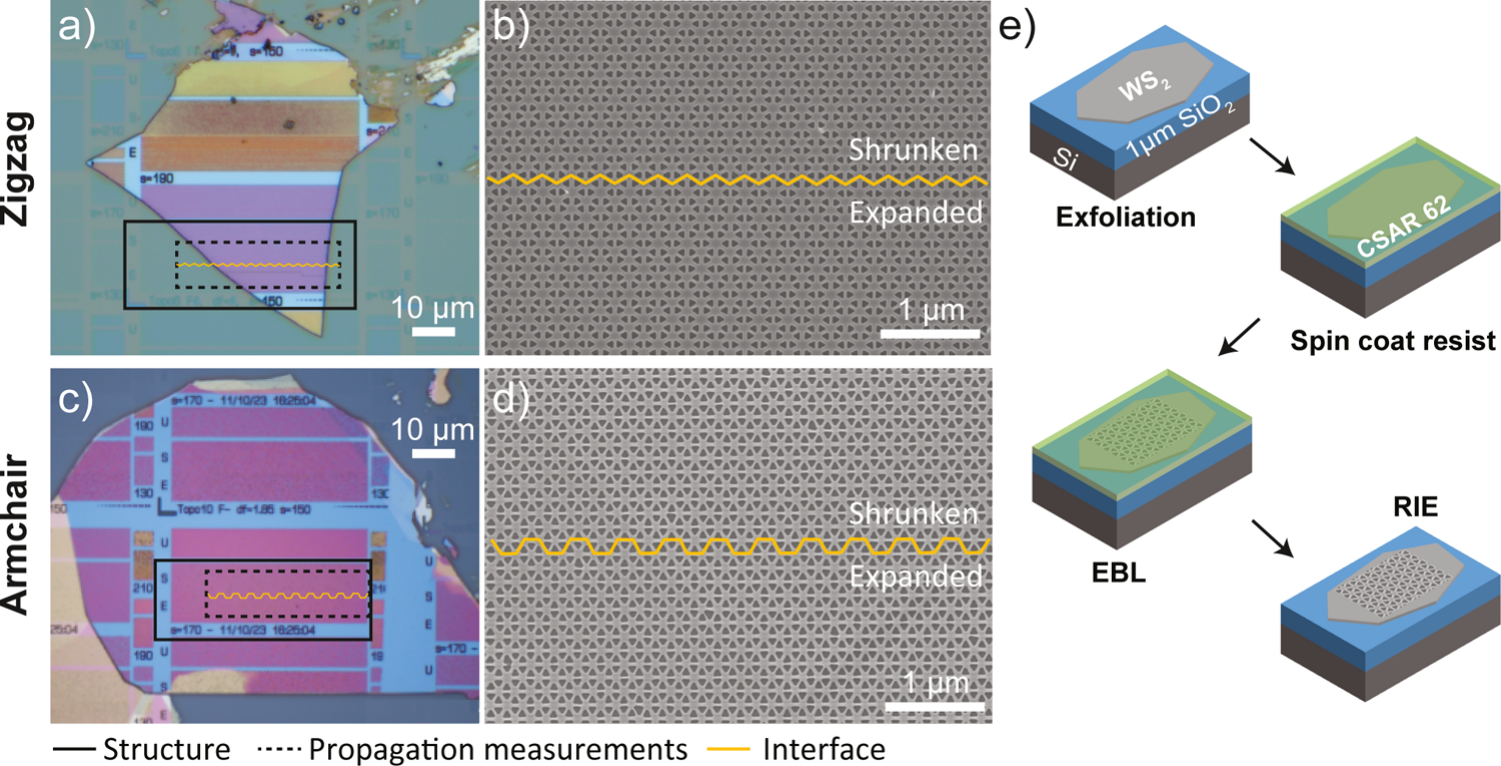
Topological structures with a lattice period *a* = 478 nm etched in a WS_2_ flake placed on a 1 μm SiO_2_/Si substrate. (a) Optical and (b) SEM images of a structure with a zigzag interface (thickness *t* = 71 nm, triangle side length *s* = 165 nm, later referred to as the zigzag sample) and (c) optical and (d) SEM images of a structure with an armchair interface (*t* = 65 nm, *s* = 185 nm, armchair sample). (e) Schematic depiction of the fabrication procedure.

Two different designs were considered: one with a zigzag domain wall between the shrunken and expanded regions (Figure 2a,b) and one with an armchair domain wall (Figure 2c,d). The slab waveguides were designed to have triangular holes of sizes ranging from 130 to 190 nm, with a period *a* of 478 nm, in a WS_2_ layer with a thickness *t* close to 70 nm, situated on top of 1 μm SiO_2_ on a silicon substrate. For the field to be satisfactorily confined within the waveguide and the selective propagation to be feasible, a minimum thickness of approximately 50 nm is needed for the WS_2_ layer, as illustrated in simulations with different thicknesses in Figure S17 of the Supporting Information.

The topological and trivial lattices were designed to have gaps at around 1.5 eV, set to be significantly lower than the main absorption peak of bulk WS_2_, associated with its direct transition near 2.0 eV.^12^ The individual photonic hole lattices are perturbed in terms of expansion (*R*_*e*_ = 1.05*R*_0_) and contraction (*R*_*s*_ = 0.95*R*_0_) of the individual hexagonal cells as detailed in Figure 1a. As shown in Figure 2e, the structures were fabricated with the use of electron beam lithography (EBL) and reactive ion etching (RIE) processes on exfoliated WS_2_ flakes (see Materials and Methods). Designs based on the equilateral triangle sides aligned with the WS_2_ crystal axis were patterned with varying doses and triangle sizes on flakes of different thicknesses. In this article, two structures with zigzag and armchair domain walls of respective triangular side lengths of 165 and 185 nm are considered, as shown in scanning electron microscopy (SEM) images in Figure 2b,d.

**Figure 3:**
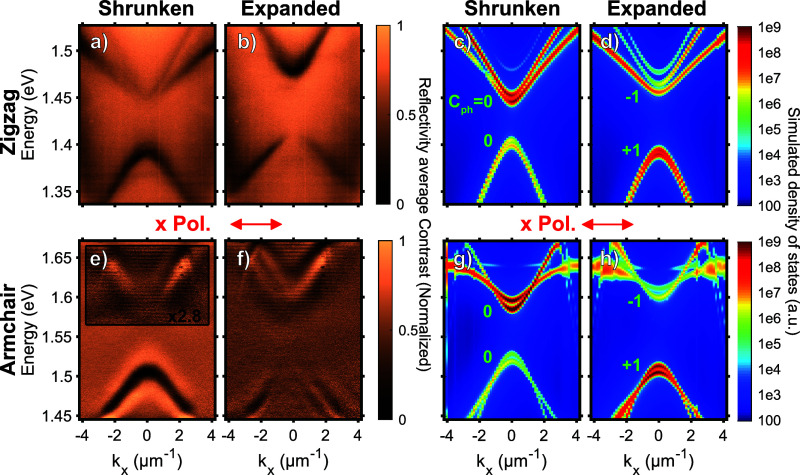
Angle-resolved reflectance contrast data for individual lattices in the zigzag WS_2_ sample (detailed in Figure 2) with (a) shrunken and (b) expanded lattice regions measured with linear polarization along the direction of the interface, *x*. (c,d) Photonic band structure simulations of the zigzag sample. (e,f) Corresponding reflectance contrast data and (g,h) simulations of the armchair sample. The spin-Chern numbers *C*_*ph*_ of the bands obtained in the simulations are indicated in the plots in green. Note that the simulations report near-field intensities in contrast to the far-field reflectance contrast spectra.

**Figure 4:**
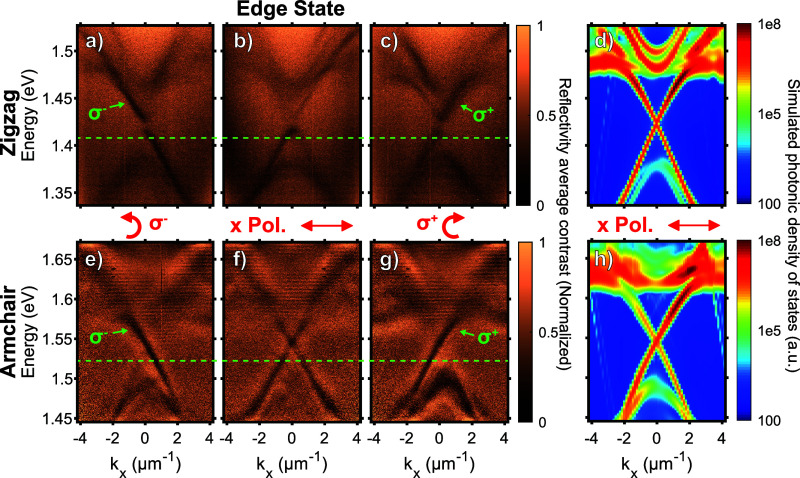
Angle-resolved reflectance contrast data for the interface between the trivial and topological regions for respectively the (a) counterclockwise polarization (σ^–^), (b) with polarization along the direction of the interface *x*, and (c) clockwise (σ^+^) polarization in the zigzag sample (Figure 2) and (d) the photonic band structure simulated for linear polarization. (e–g) Corresponding reflectance contrast data and (h) simulations for the armchair sample. The excitation wavelengths (dashed lines) used in the propagation experiments (Figure 5) and the unidirectional interface modes (marked σ^–^ and σ^+^) are highlighted in the plots.

Angle-resolved reflectivity contrast measurements shown in Figures 3 and 4 were carried out on the WS_2_ zigzag and armchair structures using spatial filtering within a Fourier-plane spectroscopy setup (more details in Materials and Methods and the Supporting Information) and were compared with FDTD simulations (see Materials and Methods).

The trivial (shrunken) and nontrivial (expanded) regions of the two structures were first considered with an incoming linearly polarized white light along the direction of the interface (*x*) (more details in the Materials and Methods section). In the case of the shrunken regions of the zigzag and armchair structures (Figure 3a,e), one can observe two parabolic dispersions: one upper mode with a positive effective mass curvature and one lower mode with a negative curvature. Moreover, the upper mode intensity vanishes for the low wavevector, while the lower mode intensity remains constant. When considering the expanded regions (Figure 3b,f), we can observe that the lower and upper bands are swapped, indicating the band inversion and the change in the topological nature of the shrunken and expanded regions. In terms of topology, the bands of the topological (expanded) lattices at the gap are known to have nonvanishing spin-Chern numbers *C*_ph_ = −1 (upper) and +1 (lower), while the shrunken lattices are topologically trivial with *C*_ph_ = 0.^8^ The dispersions from the shrunken and expanded regions are well reproduced with FDTD simulations in Figure 3c,d,g,h, with simulated parameters close to the fabricated structure dimensions. In experiments, the expanded lattice can have slightly larger triangles due to a proximity effect in exposure, which can explain the blueshift in bands in Figure 3d.

We note that due to the change of the triangle size *s*, the optical modes are located at two different spectral regions with midgap energies of 1.43 and 1.55 eV, respectively, for the zigzag and armchair structures. This shows the versatility of the studied approach employing van der Waals materials, enabling photonic crystal structures for which the spectral position of the modes can be varied by modifying the period, lattice expansion, triangle size, or the thickness of the WS_2_ flake (see Supplementary Figures S1–S3).

The interfaces between the two topologically distinct regions were then studied by collecting reflectivity signals from long and narrow rectangular regions parallel to the interfaces (*x*-axis in Figure 1b, more details in Materials and Methods). Figure 4a–c (e–g) presents the reflectance of the zigzag (armchair) structures at the interface between the trivial and nontrivial regions for respectively the counterclockwise (σ^–^), *x*-linear as in Figure 1b, and clockwise (σ^+^) polarizations. In the case of the *x*-linear polarized light (Figure 4b,f), we can observe two modes with linear dispersions with slopes of opposite signs occurring within the gap between the two parabolic photonic modes of the shrunken and expanded regions described previously. These linear modes are the counter-propagating edge state modes occurring at the interface between the trivial and nontrivial regions whose propagating directions are given by the signs of their slopes. To confirm that these modes are indeed edge states, we performed the same reflectivity contrast measurements for the counterclockwise (σ^–^) and clockwise (σ^+^) polarizations (see Figure 4a,c,e,g). We observed that only the edge-state mode with a positive (negative) slope occurs for the counterclockwise (clockwise) polarization. This means that we can select the propagation direction of the edge state mode by switching from counterclockwise to clockwise polarization.^33,35^

One can notice that in the case of the zigzag structure, a small gap is opened at the intersection between the two edge state modes (see Figure 4b). This gap is also visible when the edge state modes are isolated in the counterclockwise and clockwise polarization (see Figure 4a,c). This small gap is seen in some cases due to broken crystal symmetry at the interface^33,36^ and has been attributed to spin–spin scattering in recent studies indicating intrinsic limits of topological protection.^27,29^

**Figure 5:**
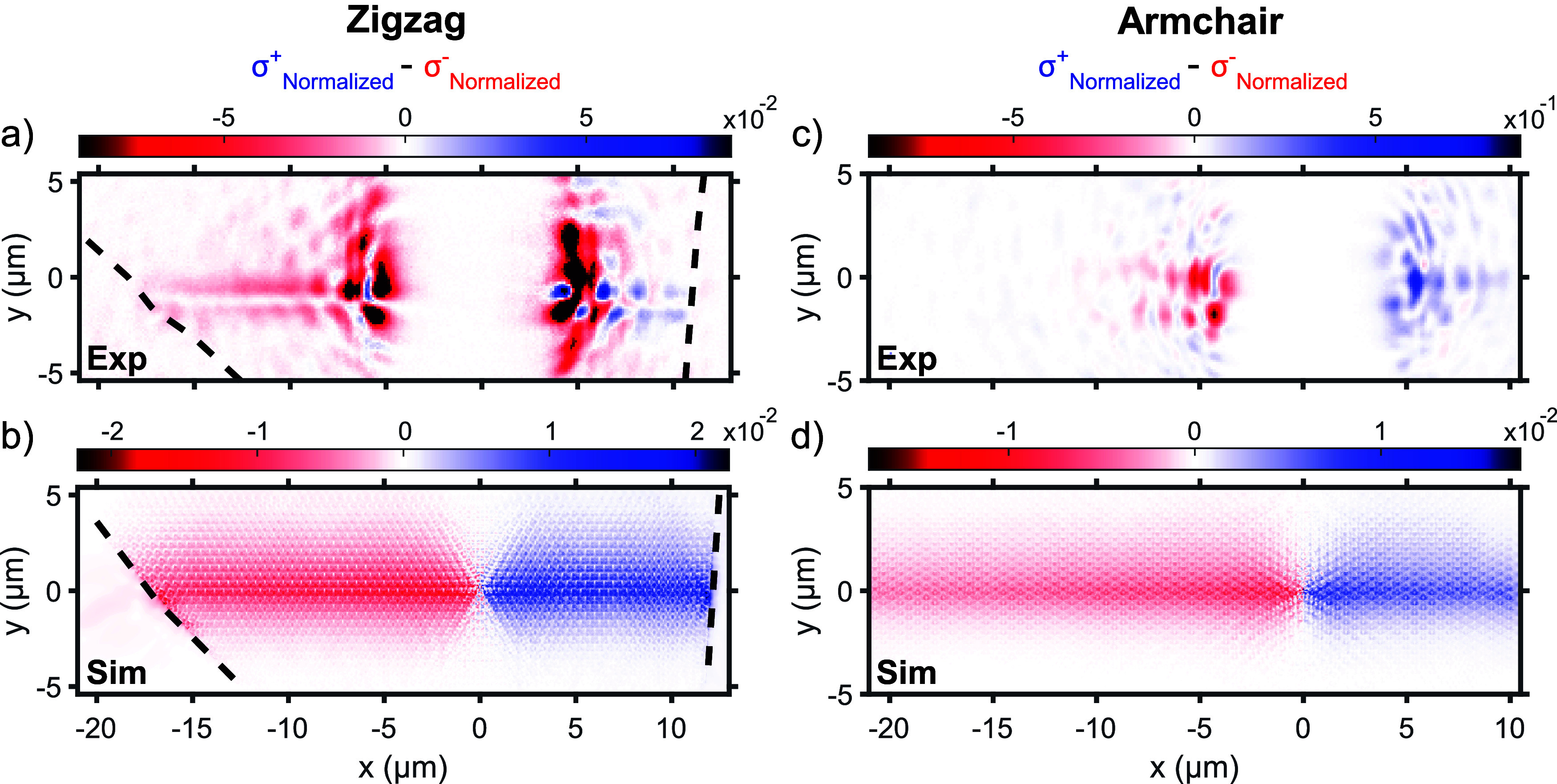
Light propagation along the domain boundary in the spin-Hall WS_2_ structure. Experimental intensity differences at TE (E field in plane) polarization for (a) zigzag and (c) armchair structures. The min-max normalized clockwise (σ^+^) signal (normalized between minimum and maximum values) is subtracted from the min-max normalized counterclockwise (σ^–^) one. (b,d) Corresponding differences of the simulated electric field in the zigzag and armchair structures. The black dashed lines in (a) and (b) indicate the border of the flake of the zigzag structure (see Figure 2a). The white area in the middle of the experimental signals corresponds to the spatial filtering used to suppress the direct intense reflection from the input laser.

The propagation of the edge states at the topological interface of both structures has been further studied both experimentally and in simulation. The zigzag and armchair topological structures were resonantly excited with an input laser at 881 nm (1.41 eV) and 815 nm (1.52 eV), respectively, within the gaps of the band structures (see arrows in Figure 4). The areas where the propagation was studied experimentally for both structures are shown in Figure 2a,c. The laser was focused in the middle of these areas at the border between the shrunken and expanded regions. The scattered light from the excitation point was then collected with the Fourier spectroscopy setup and projected to the spectrometer CCD camera. In order to suppress the direct intense reflection from the input laser, the scattering signal was filtered in real space with the use of a wire placed on a lens mount (more details are in the Materials and Methods section). Using this technique, long exposure times could be used to observe the emitted light away from the excitation point. The intensity difference between the normalized signals of the clockwise (σ^+^) and counterclockwise (σ^–^) TE (E field in plane) circular polarizations is obtained in this way and is shown in Figure 5. The signals for both polarizations are shown in Figure S10 of the Supporting Information.

Figure 5a,b shows the experimental and simulated intensity differences (σ^+^ – σ^–^) of the zigzag structure with the propagation stopping at flake edges (black dashed lines, see Figure 2a). The degree of circular polarization ((σ^+^ – σ^–^)/(σ^+^ + σ^–^)) is shown in Figure S15 of the Supporting Information. In the experiment, we observe a strong scattering from the laser with a white strip in the middle corresponding to the spatial filtering mentioned previously. However, away from the directly scattered signal, one can observe signal stemming from unidirectional propagating modes, propagating leftward for counterclockwise polarization (in red) and rightward for clockwise polarization (in blue) and in both directions for linear polarization. Indeed, as expected, propagation is blocked at the gap for the shrunken and expanded lattices but is allowed at the interface.

For the counterclockwise polarization, the propagation length was estimated to be of 9 and 50 μm, respectively for the experiment and simulations (see Figures S11 and S12 of the Supporting Information), which is comparable to the 11 μm reported on a silicon-on-insulator structure with a similar design.^31^ In the simulation, vertical sidewalls are assumed, and inconsistencies in the shape and size of different holes are not accounted for, which would explain the increased distance in propagation. The selectivity ratio between the polarizations is 23 in the simulation, showing good unidirectionality for the interface modes.

In simulation, we also consider the same structures with bends in the topological interfaces (see Figure S14 in the Supporting Information). Moreover, we found that the effect of the substrate on the propagation length is relatively small, as the same simulated structure, but suspended, sustains a propagation length of 64 μm (see Figure S16 of the Supporting Information). Further improvement for the propagation length in the future could be to consider a gradual interface rather than a step border between the shrunken and expanded areas, as it has been shown to drastically increase the propagation length.^31^

Similar results are observed from the armchair structure (see Figure 5c,d), but with much smaller propagation lengths, which limits the good quantitative observation of the propagation. This difference in the propagation lengths is attributed to the reduced quality of the structure and unoptimally large triangle holes achieved in fabrication for the case of the armchair structure compared to the zigzag structure. Nevertheless, the observation of the edge state modes within the gap of the photonic band structure in reflectance measurements and the direct experimental observation in real space of the selective unidirectional propagation, both well reproduced with FDTD simulations, confirm the topological nature of propagation at the interface of the WS_2_-based spin-Hall lattice domains.

## Conclusions

In conclusion, we have demonstrated the feasibility of using bulk TMD structures for photonic topological insulators with their associated unidirectional interface states. The fabricated insulators have a clear bandgap seen in reflection, and the interface states can be selected with the handedness of circular polarization. The selectiveness in propagation is clearly seen, with a decay length of 9.2 μm measured for the zigzag σ^–^ interface mode compared with 50 μm when the structure is simulated. This initial demonstration uses a spin-Hall design with modes at Γ point above the light cone for ease of measurement, and as such, the propagation is lossy. Using valley-Hall lattices and separate outcouplers in further experiments will substantially improve these metrics.

Our results emphasize favorable properties of quasi-bulk TMDs and more generally layered van der Waals materials and their suitability for the fabrication of complex photonic structures. We demonstrate that similarly to silicon-on-insulator structures and in contrast to GaAs topological photonic crystals, we did not have to rely on suspended membranes for achieving the high refractive index contrast necessary for optical confinement. Going forward, van der Waals materials are an attractive option for hybrid topological photonics relying on heterointegration using pick-and-place nanofabrication approaches.

## Materials and Methods

### Simulations

Finite difference time-domain simulations of the photonic structures were performed with Lumerical. The anisotropic refractive index functions for bulk WS_2_ used in simulations were obtained with ellipsometry.^12^ The dimensions of the structures on the sample used for the simulations are detailed in Figure 2. The triangles are truncated in the simulations (see Figure S3 in the Supporting Information) to match the imaged structures with the chamfer values *c* = 0.16 for the zigzag and *c* = 0.12 for the armchair sample. For band structure simulations, a 3D model of a doubled hexagonal unit cell was used since Lumerical requires the repeated Bloch cell to be rectangular. An array of dipoles matched to the unit cells was used with time monitors of the electric field, recording intensity at different frequencies. Simulations with a sweep of different momentum values were done to map out the band structure of the structures, running for a time of 3000 fs. Bands at Γ point were simulated with sweeps of various parameters. Edge mode band structure simulations were performed with a supercell containing the interface of topological and trivial lattices (a strip of 9 unit cells of each), using Bloch edge conditions in the direction of the interface and perfectly matched layers in other directions.

Propagation with chiral polarization was simulated by using a perpendicular pair of dipole sources with a 180° phase difference between them. The simulation area was 10 μm by 32 μm, which also included the edges of the flake for the zigzag sample. The model was run for 400 fs in the σ^+^ and 700 fs in the σ^–^ case, and the electric field intensity was integrated over that time. The propagation times were selected so that the propagation reaches the edge of the flake or the photonic crystal so that the propagation length fit is from one pass and that it is not overestimated by any possible reflection.

### Fabrication

The WS_2_ topological structures are fabricated on silicon substrates purchased from Inseto with 1 μm of thermally grown silicon dioxide. First, the substrate is cleaned by acetone (10 min) and isopropanol (IPA) (10 min) in an ultrasonic water bath and then blow-dried with nitrogen; after that, we treat the substrate in oxygen plasma to remove the residues and contaminants. During the cleaning procedure, WS_2_ flakes were prepared by the mechanical exfoliation method, repeatedly cleaving with a PVC semiconductor wafer processing tape, on commercially available WS_2_ crystals (HQ Graphene, synthetic), and then transferred on the clean substrate right away.^37^ The thickness of the flake was confirmed by atomic force microscopy (AFM Dimension Icon). After we find flakes close to the target thickness (70 nm), we check the candidate flakes by a 100× microscope to make sure the surface of the flake is uniform and flat and measure the crystal axis of the flake compared to the bottom edge of the substrate. See Figure S13 in the Supporting Information for an illustration of the alignment and a comparison between an unoptimized structure and the structures used in the measurements.

Second, the sample is prepared for EBL. The spin coating procedure consists of two steps, each involving the deposition of the respective film and successive baking on a hot plate. First, spin coating the positive electron-beam resist CSAR 62 (AR-P 6200.13) of thickness 350 nm is then covered with a conductive layer of Electra 92 (AR-PC 5090.02) to mitigate charging effects that could reduce the patterning resolution during EBL. In the EBL step, the topological structure design is patterned into the resist layer using an EBL machine (Raith VOYAGER). The pattern for EBL has exaggerated pointed triangles to improve the resulting shape of the holes in the photonic crystal. The pattern is oriented so that the triangle sides of the pattern coincide with the zigzag crystallographic edge of the individual WS_2_ flake to take advantage of the anisotropic dry etch in improving the shape of the triangle. To determine the optimum exposure dose, a dose test is typically performed prior to the fabrication of the actual sample layout. This entails patterning multiple copies of a test design, each with slightly different exposure doses, which are then checked with an SEM to find the optimum value.

The EBL step size was 4 nm, which is the resolution limit for the individual structures in our samples. As *R*_0_ = 159.3 nm, the difference between shrunken and expanded lattices is 16.0 nm, significantly larger than the step size. Based on SEM images, the position repeatability over a 10 μm distance is better than 0.2% or ±2 nm for the period a = 478 nm, similar to the scanning accuracy of the SEM. In our experiments, the pattern is small and within the same 100 μm writefield, so any drift (specified as <120 nm over 8 h) does not seem to affect the positioning, and stitching errors are not applicable. Additionally, the proximity effect of the exposure (due to electron scattering from the substrate) will shift the effective position of the triangles depending on the exposure dose. Based on the simulations of the effects of changing the radius of the triangle position (plotted in Figure S2 in the Supporting Information), we are also confident that the positioning error is effective at the scale of 4 nm or less.

After the patterning process, the sample is immersed in DI water to remove the layer of Electra 92, then in xylene to dissolve the exposed areas of the positive resist CSAR 62, and finally in IPA to get rid of chemical residues prior to blow-drying. The patterned layer of resist covering the sample surface acts as a mask for the subsequent RIE, upon which the design is transferred into the WS_2_ flake. Before etching our sample, we cleaned the chamber with Ar + H_2_ and O_2_ plasma. We used a combination of CHF_3_ and SF_6_ plasma to provide a mixture of physical and chemical etching that gives us the best topological structures. After successful RIE, the residual resist film is removed by immersion in a hot 1165 resist remover (90 °C, 30 min) and hot acetone (90 °C, 30 min), followed by a rinse with IPA and several seconds of O_2_ plasma ashing to remove the harder resist residues caused by RIE.

### Optical Measurements

Angle-resolved reflectivity contrast measurements shown in Figures 3 and 4 were carried out using a spatial-filtering in a Fourier spectroscopy setup (see Figure S4 in Supporting Information). The sample is illuminated in a large region by a collimated white light using a 0.7 NA objective (100X Mitutoyo Plan Apo NIR) and a 150 mm lens before the objective. The reflected light is collected by the same objective and is separated from the input signal with a beam splitter. The image of the sample is then projected by the objective and a 250 mm lens onto a double slit which selects the desired rectangular region of the sample. A 600 mm lens placed at the focal length behind the spatially filtered real space performs the Fourier transform of the signal. The Fourier space located at the focal length behind the 600 mm lens is then projected with a set of two lenses (200 mm, 150 mm) onto the slit of a spectrometer which selects the wavevectors along the vertical direction. The diffractive grating inside the spectrometer disperses the light horizontally, and the signal is projected onto a 400 × 100 CCD camera, resulting in a (*k*_*x*_,λ) reflectivity dispersion signal from the rectangular region of interest.

In the case of the measurements of the trivial (shrunken) and nontrivial (expanded) regions, the reflectivity measurements were done using a large rectangular region on the structures (see Figure S5 in the Supporting Information). In the case of the measurements at the interface of the two distinct topological regions, the signals were taken from a long and thin rectangular region, parallel to the interface, in order to isolate the signal from the edge states (see Figure S5 in the Supporting Information). For each measurement of the structures, another measurement was taken from the unpatterned part of the WS_2_ flake as a reference in order to perform the reflectivity contrast processing.

The real-space propagation measurements were performed using the same Fourier spectroscopy setup in which the last two lenses were replaced by a 700 mm lens (see Figure S9 in the Supporting Information). This large focal lens permits one to project the real space image with a larger magnification to the CCD camera instead of the Fourier space in the case of the dispersion measurements. The photonic structure was excited with a filtered output from a supercontinuum laser whose wavelengths were put in resonance with the edge state modes. A wire placed on a lens mount was placed in the first real space after the objective (see Figure S9 in the Supporting Information) in order to spatially filter the direct reflection of the input laser. Using this technique, long exposure times could be used to observe the scattered light away from the excitation point.

### Data Processing

In the case of the reflectivity measurements on the zigzag structure, the reflectivity contrast signal considered was the subtraction of the structure signal by the unpatterned flake signal (*S* = *S*_structure_ – *S*_reference_). However, in the case of the armchair structure, a parasitic Fabry–Perot resonance from the thick SiO_2_ layer hindered the visibility of the optical modes. The signals from the structure and the unpatterned part of the structure were fitted at each *k*_*x*_ slice with a Gaussian line corresponding to the Fabry–Perot mode, which was then removed from the signals. Both the signals were then smoothed in a similar fashion as in ref (8) before performing the same treatment as previously (). More details of the data treatment are given in Supplementary Figures S6–S8.

## Supporting Information Available

Calculated dependence on thickness, expansion factor, triangle side and corner chamfer of the TE-like photonic band gaps of the WS_2_ lattices at Γ; angle-resolved reflectivity contrast measurement method on a spatial-filtering Fourier setup; method of the band structure measurements of the interface and the shrunken and expanded lattices; data treatment of the band structure reflectivity measurements; Fabry-Perot mode data treatment method; average contrast treatment method; propagation measurement method; experimental and simulated propagation results; propagation length determination from the simulations and the measurements; difference in fabrication resulting from optimization and alignment to the WS_2_ crystal structure; simulated propagation along a meandering topological interface; degree of circular polarization in propagation; simulated effect of the substrate and the waveguide thickness on the propagation (PDF)

## Notes

A previous version of this work has been uploaded to a preprint server: Tommi Isoniemi; Paul Bouteyre; Xuerong Hu; Fedor Benimetskiy; Yue Wang; Maurice S. Skolnick; Dmitry N. Krizhanovskii; Alexander I. Tartakovskii. Realization of Z2 topological photonic insulators made from multilayer transition metal dichalcogenides. 2024, arXiv: 10.48550/arXiv.2407.05908 (accessed October 15, 2024)
