# Supplementary material for: Realization of Z2 Topological Photonic Insulators Made from Multilayer Transition Metal Dichalcogenides
Source: ACS Nano. 2024 Nov 18;18(47):32547–55. doi: 10.1021/acsnano.4c09295 (PMC11603781; doi:10.1021/acsnano.4c09295)
Supplement: Supplementary file 1 — nn4c09295_si_001.pdf [file nn4c09295_si_001.pdf]

Supporting Information: Realization of Z_2 topological photonic insulators made from multilayer transition metal dichalcogenides

Tommi Isoniemi,[1, a)] Paul Bouteyre,[1, b)] Xuerong Hu,[1, c)] Fedor Benimetskiy,[1] Yue Wang,[2] Maurice S. Skolnick,[1] Dmitry N. Krizhanovskii,[1] and Alexander I. Tartakovskii[1, d)]

[1)] *Department of Physics and Astronomy, University of Sheffield, Sheffield S3 7RH, U.K.*

[2)] *School of Physics, Engineering and Technology, University of York, York YO10 5DD, U.K.*

(Dated: 15 November 2024)

[a)] Contributed equally to this work as first authors; Electronic mail: t.isoniemi@sheffield.ac.uk

[b)] Contributed equally to this work as first authors; Electronic mail: p.bouteyre@sheffield.ac.uk

[c)] Contributed equally to this work as first authors

[d)] Electronic mail: a.tartakovskii@sheffield.ac.uk

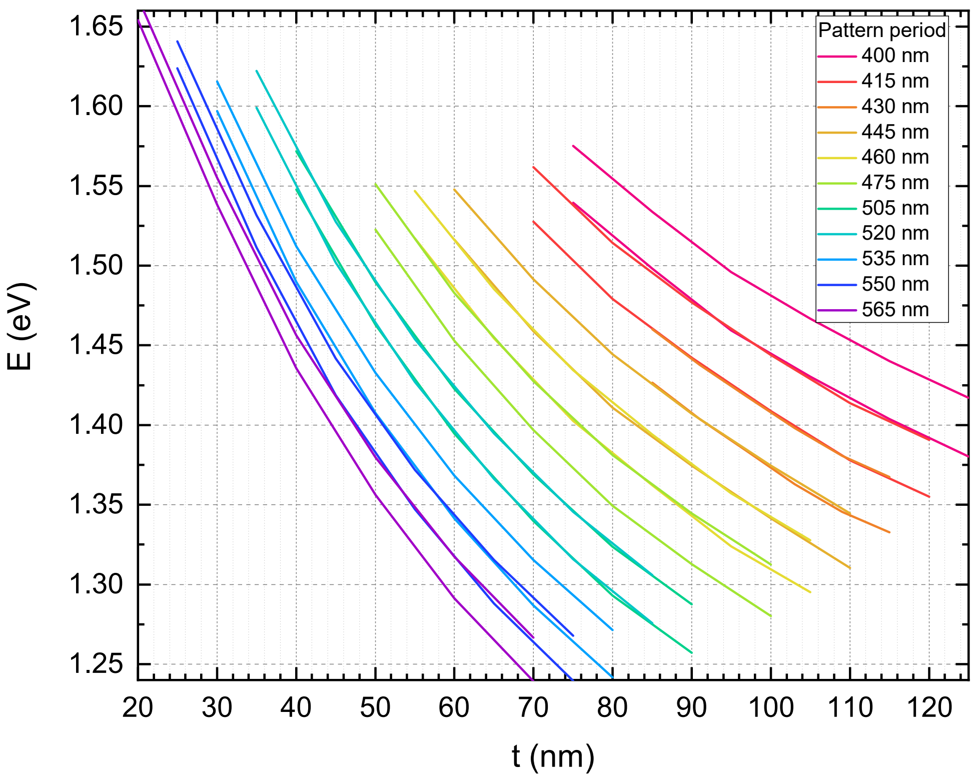

FIG. S1. Calculated TE-like photonic band gaps at Γ of a shrunken (0.95) hole lattice of triangles with their side length kept at $s = 0.315a$. The thickness t of the WS_2 flake is varied on a 1 μm SiO_2/Si substrate. Lines with different colours correspond to different periods a.

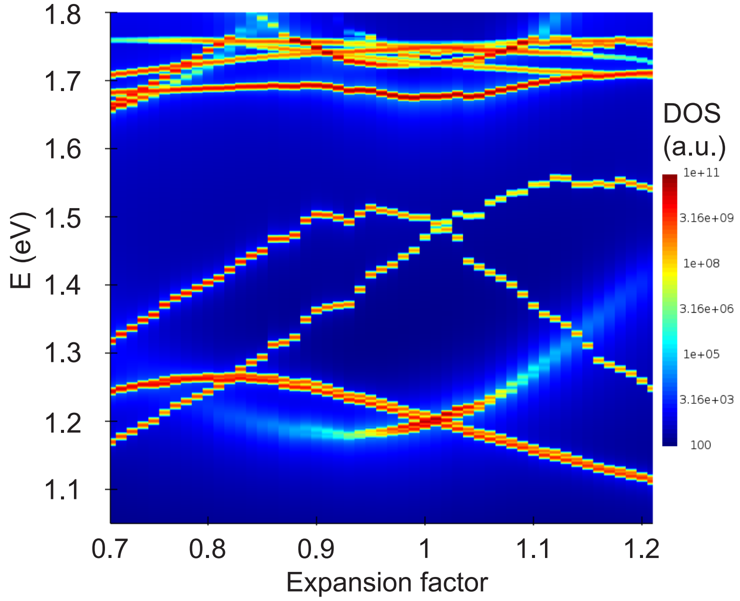

FIG. S2. Calculated TE-like photonic bands of spin-Hall lattices in WS_2 at Γ with various expansion factors. The relevant bands cross at 1.2 eV at factor 1. a = 560 nm, h = 80 nm, c = 0.

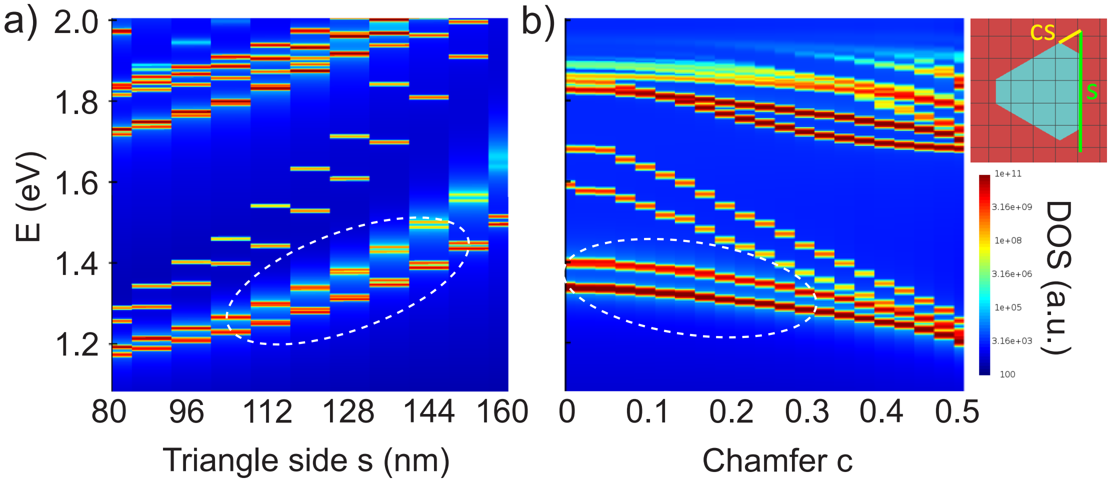

FIG. S3. Calculated TE-like photonic bands at Γ. a = 440 nm, h = 100 nm, expansion factor 1.05. a) Triangle size (with c = 0) is changed. The energy of the bands increases as the triangle holes increase in size and the effective refractive index of the film decreases. b) Cutoff of the triangle corners (with s = 138 nm) is varied. In both cases the relevant gap is highlighted. The chamfer parameter is defined with the distance cut from every corner of each triangle. Illustration of a triangle hole with a chamfer value c = 0.2 is shown in the inset.

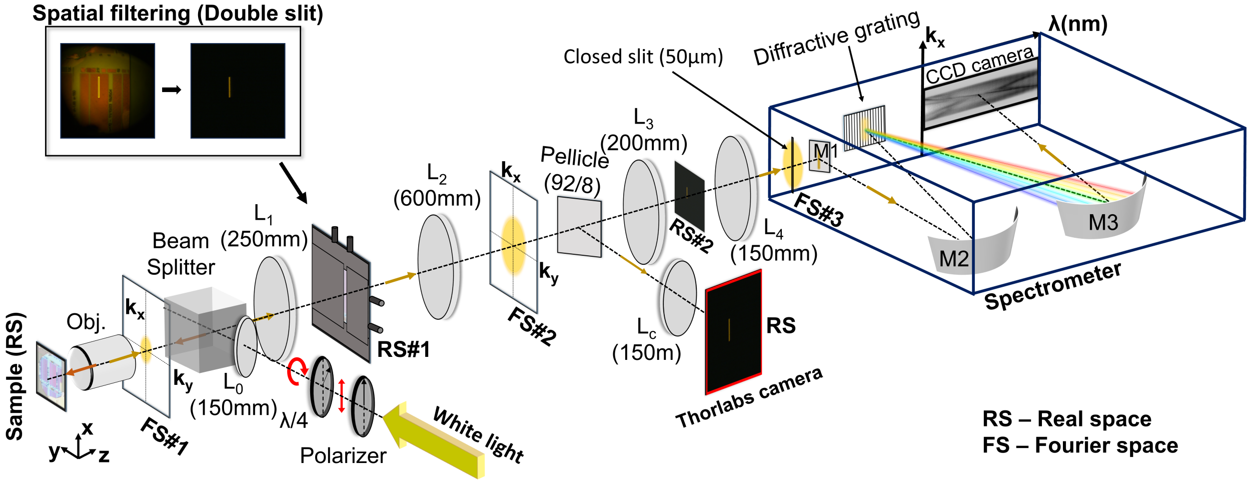

FIG. S4. Angle-resolved reflectivity contrast measurements were carried out using a home-made spatial-filtering Fourier set-up. The sample is illuminated in a large region by a collimated white light using a 0.7 NA objective (100X Mitutoyo Plan Apo NIR) and a 150 mm lens (L_0) before the objective. The reflected light is collected by the same objective, and is separated from the input signal with a beam splitter. The image of the sample is then projected by the objective and a 250 mm lens (L_1) onto a double slit which selects the desired rectangular region of the sample. A 600 mm lens (L_2) placed at focal length behind the spatially-filtered real space performs the Fourier transform of the signal. The Fourier space (#2) located at focal length behind the 600 mm lens is then projected with a set of two lenses (200 mm (L_3), 150 mm (L_4)) onto the slit of a spectrometer which selects the wavevectors along the vertical direction. The diffractive grating inside the spectrometer disperses the light horizontally and the signal is projected onto a 1340x400 CCD camera resulting in a (k_x,λ) reflectivity dispersion signal from the rectangular region of interest.

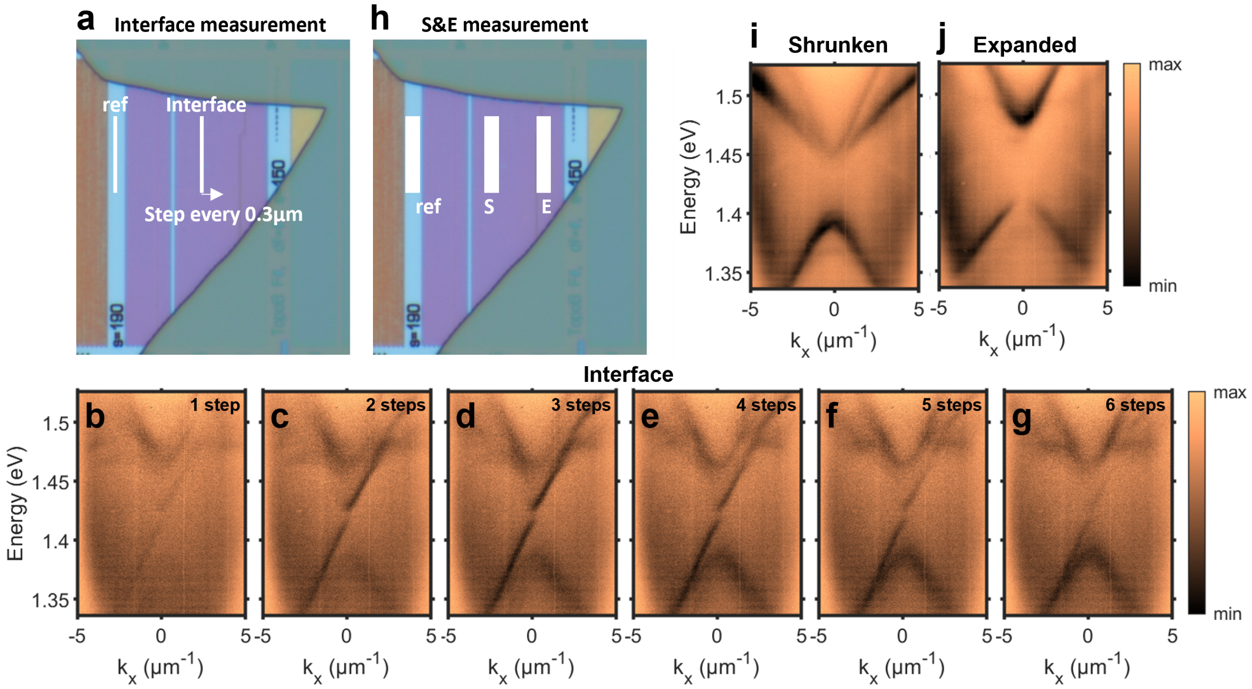

FIG. S5. Method of the band structure measurements of the interface and the shrunken and expanded lattices. (a) Interface measurement method. An 1x20 μm^2 rectangular region is selected with the double slit placed in the real space (see Figure S4). The reflectivity measurements were first performed on the unpatterned part of the flake as reference signal, and several ones taken 16.8 μm + nx0.3 μm steps on the right. The resulting results at different steps are shown in (b) to (g). This method was used to obtain the best region of the interface modes. (h) Shrunken and expanded lattices measurement method. A bigger rectangular region is considered (2.5x20 μm^2), and the resulting results are shown in (i) and (j). The treatment method of this measurement are further detailed in Figures S6, S7, and S8.

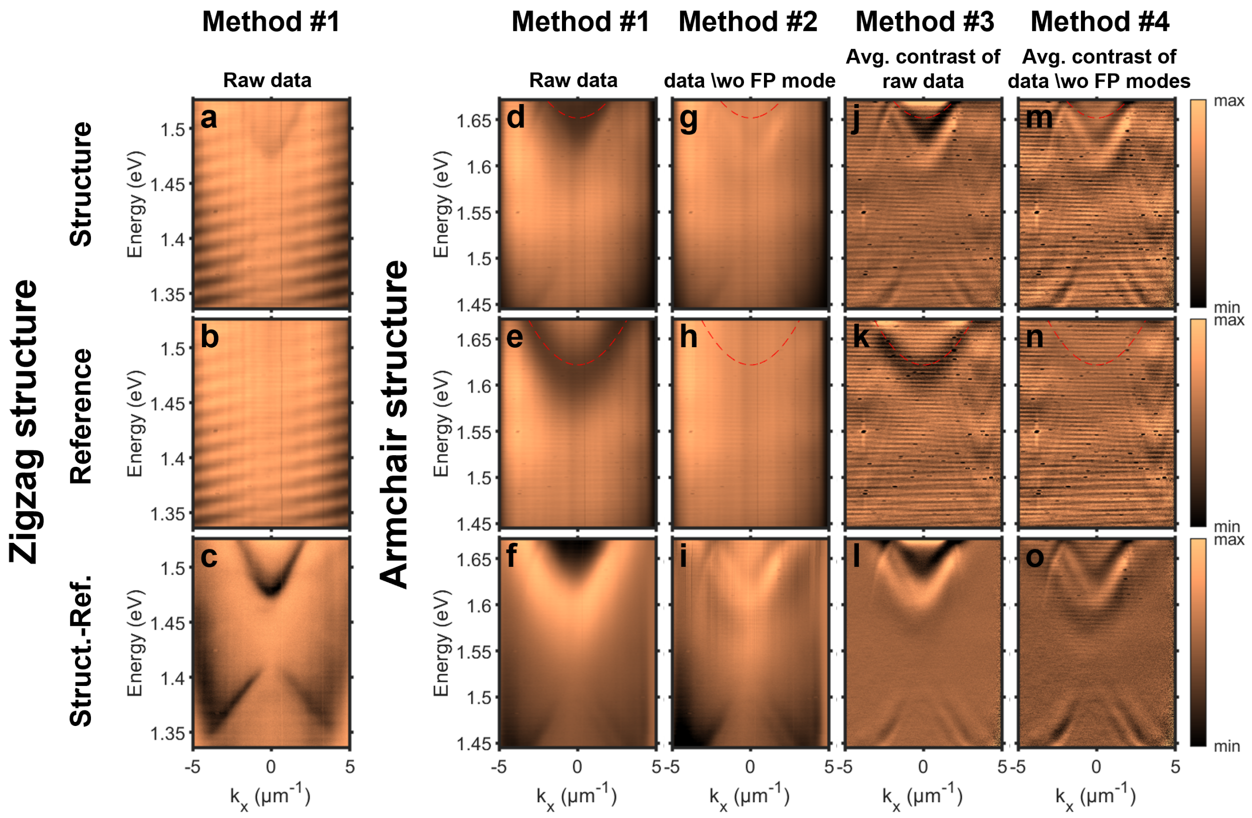

FIG. S6. Data treatment of the band structure reflectivity measurements. a) to c) reflectivity measurement of the zigzag structure expanded region with (a) the structure reflectivity measurement, (b) the flake reference measurement and (c) reflectivity difference between the structure and reference measurement. d) to f) reflectivity measurement of the armchair structure expanded region. In this case, a Fabry-Perot (FP) mode is localized directly on top of the expanded top mode. Moreover, the FP mode is redshifted between the structure (d) and reference (e) measurements as the two regions possess slightly different effective refractive index. When plotting the reflectivity difference (f), the two shifted FP modes are visible, which hide the expanded upper mode. g) to i) Structure (g) and reference (h) signals where the FP modes were fitted and removed (see method in Figure S7). The upper mode of the photonic band structure is visible in differential reflectivity signal (i), but with low contrast. j) to l) Structure (j) and reference (k) signals smoothed in a similar fashion as in[1] (see method in Figure S8). The differential reflectivity (l) possess a better contrast but the upper mode is still hidden by the FP modes. m) to o) Structure (m) and reference (n) signals for which the FP modes were removed and then smoothed. The differential reflectivity (o) possess a good contrast and upper mode visibility using the combination of the FP mode removal technique and smoothing.

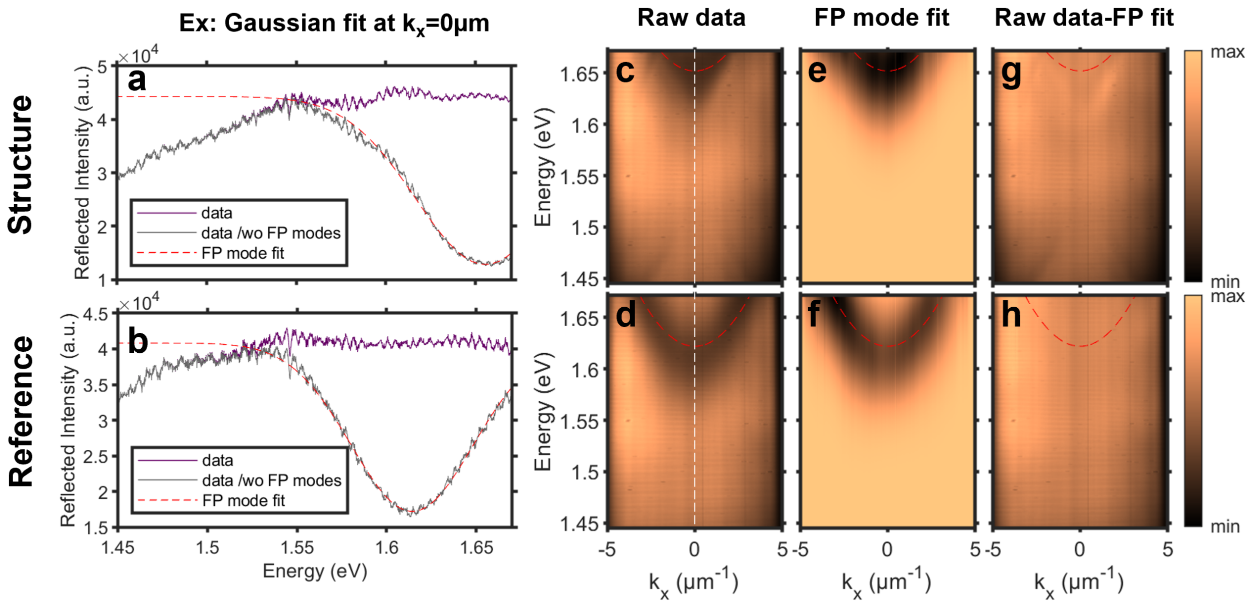

FIG. S7. Fabry-Perot (FP) mode fitting method for the armchair structure measurements. Each vertical slices at given k_x of the reference (c) and structure (d) raw data measurement were fitted with a Gaussian line to remove the Gaussian FP resonance. Examples of these fits for $k_x = 0$ are shown for the reference (a) and structure (b) signals. The resulting FP mode fitting for all the vertical slices are shown in the colormaps in (e) and (f). The difference between the raw data and the fitted FP modes are shown respectively in (g) and (h) for the reference and structure raw data.

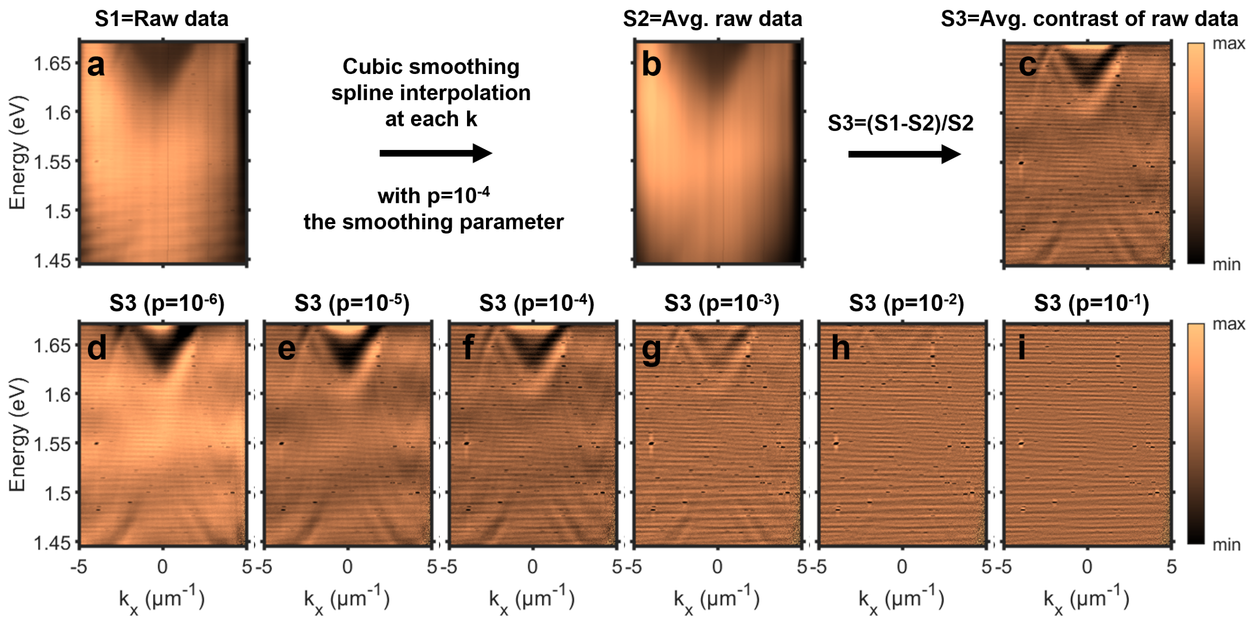

FIG. S8. Average contrast treatment method. The raw data signals, S1 (a), is smoothed, S2 (b), using the cubic smoothing spline interpolation Matlab function with a smoothing parameter $p = 10^{-4}$. The reflectivity contrast S3=(S1-S2)/S2 is shown in (c). (d) to (i) Average contrast treatment method, for smoothing parameters ranging from 10^{-6} to 10^{-1}. When the smoothing parameter p is too small, $p < 10^{-4}$, the Fabry-Perot (FP) mode is not entirely removed. When the smoothing parameter is too high, $p > 10^{-4}$, the FP mode is well removed but also the structure optical bands. For the armchair structure it was found that the parameter $p = 10^{-4}$ combined with a FP mode removal detailed in Figure S7 was the optimal method to remove the FP mode as much as possible while keeping the structure optical modes.

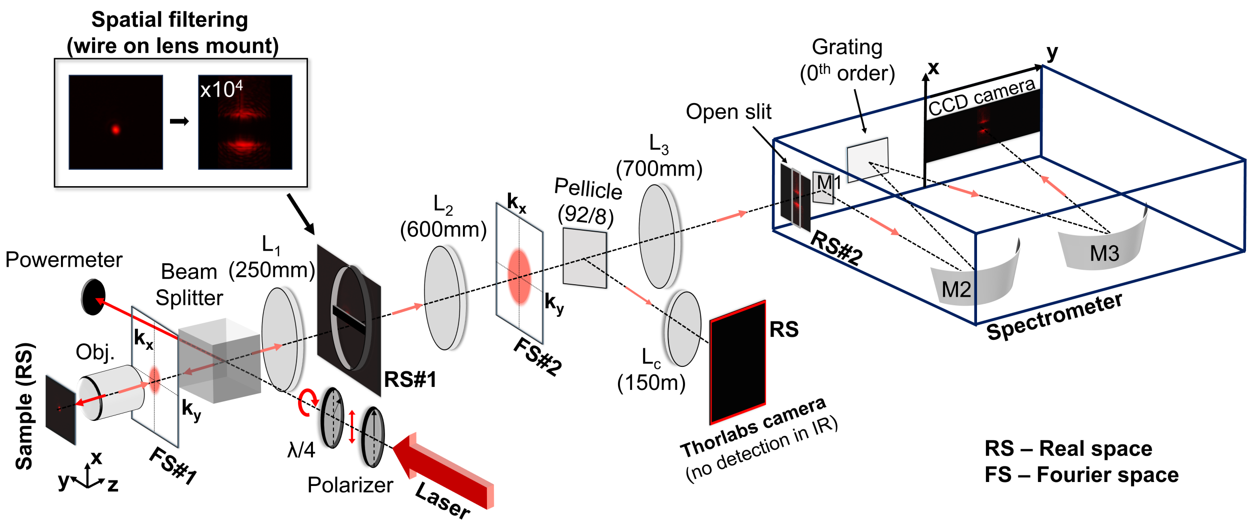

FIG. S9. Propagation measurements were carried out using a slightly altered Fourier setup where the Fourier lens L3 and spectrometer lens L4 were flipped down and replaced by a spectrometer lens L5 of large 700 mm focal length in order to get a large real space image onto the CCD camera. The sample image (real space) is projected a first time with the 100x objective and the 250 mm lens. We proceed to a spatial filtering by placing a wire on the first projected real space. This is to get rid of the intense laser signal at the excitation spot in order to see the propagation signal away from the laser spot. Then, the filtered real space is projected to the spectrometer and goes through the spectrometer slit and is projected onto the CCD camera as the spectrometer grating is put at 0th order and acts as a mirror.

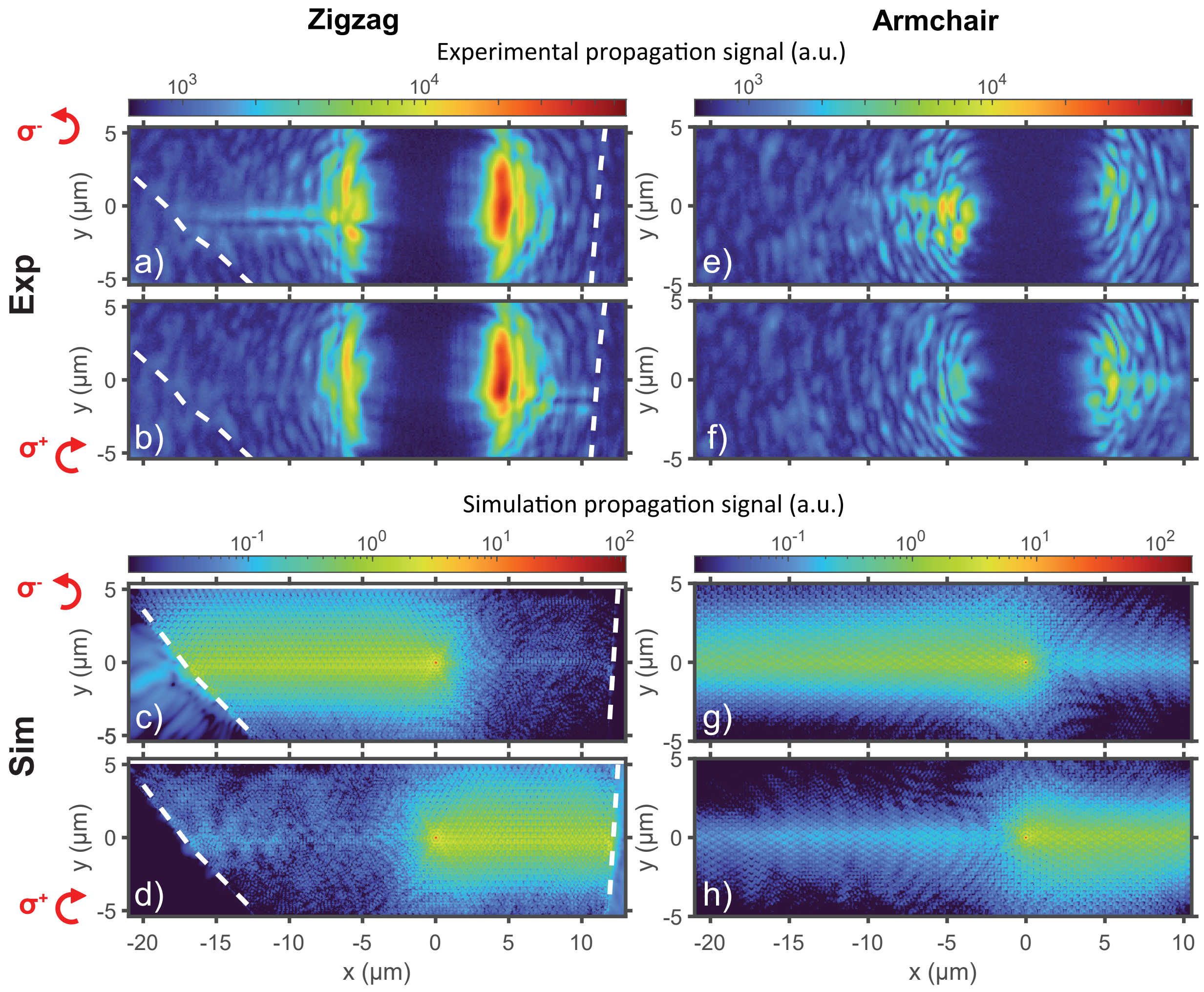

FIG. S10. Scattered light coming from the zigzag and armchair structures using the propagation setup in S9. a) and b) (e) and f)) scattered signal from the zigzag (armchair) structure for respectively the counter-clockwise circular and clockwise circular TE (E field in plane) polarizations. The corresponding FDTD simulations with integrated E field at the center plane of the WS_2 layer are shown in c) and d) (g) and h)). The white dashed lines in a-d) indicate the border of the flake in the case of the zigzag structure (see Fig. 2a) in the article). The data used is the same as in Fig. 5 of the article.

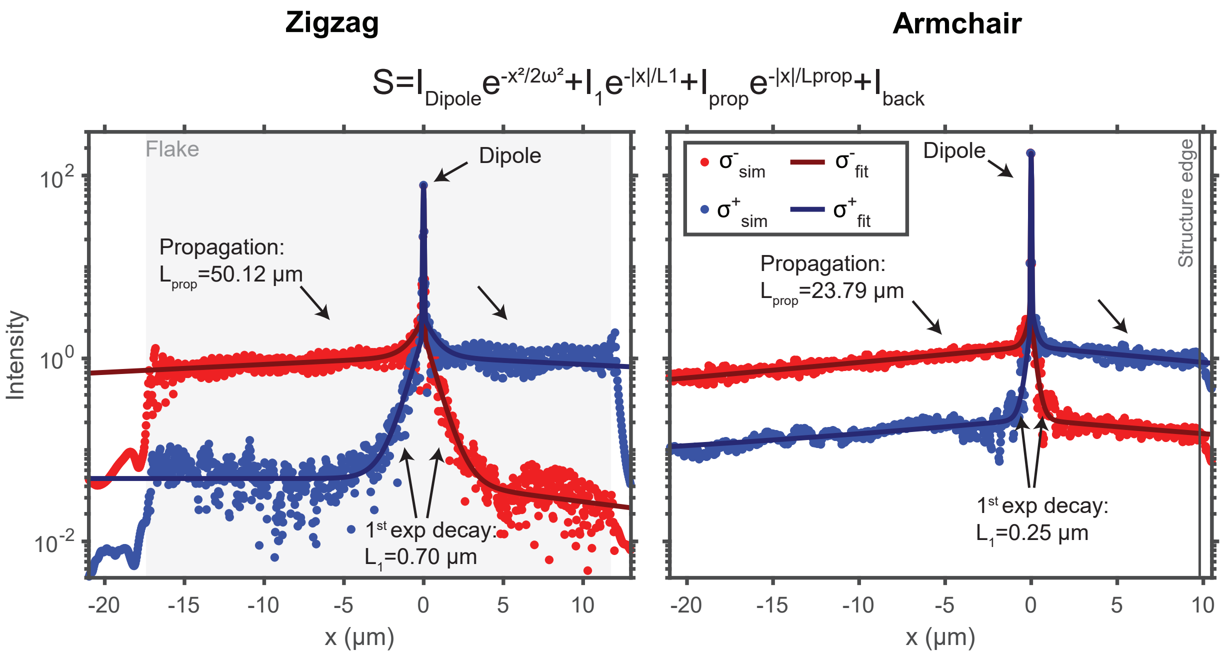

FIG. S11. Propagation lengths from the simulation of the zigzag and armchair structures. The cross-sections taken from the simulations shown in Figures S10c-d) and g-h) were taken at $y = 0$ µm. The cross-sections of the counter-clockwise polarization simulations (σ^-, Fig. S10a)) are plotted as red dots, and the clockwise polarization (σ^+, Fig. S10b)) as blue dots. Both cross-sections were fitted separately for positive and negative x, with the following equation: $S = I_{Dipole}e^{-x^2/2\omega^2} + I_1 e^{-|x|/L_1} + I_{prop}e^{-|x|/L_{prop}} + I_{back}$, with I_{Laser}, I_{prop}, I_1, I_{back}, the dipole, first decay, propagating mode, and background intensities, ω the dipole waist, and L_{prop} the propagation length. Propagation lengths of 50.12 µm and 23.79 µm were found for respectively the zigzag and armchair structures. One can notice that there is still propagation along the interface for σ^+ (blue) polarization for negative x, and σ^- (red) polarization for negative x. However, the intensity of these propagation are an order of magnitude lower than for the opposite circular polarization. The directional selection power, corresponding to the signal ratio between the propagation in one direction of the two circular polarizations, $I^{\sigma^-}_{prop(left)}/I^{\sigma^+}_{prop(left)}$ or $I^{\sigma^+}_{prop(right)}/I^{\sigma^-}_{prop(right)}$, are 23.34 and 8.02 for the zigzag and armchair structures, respectively.

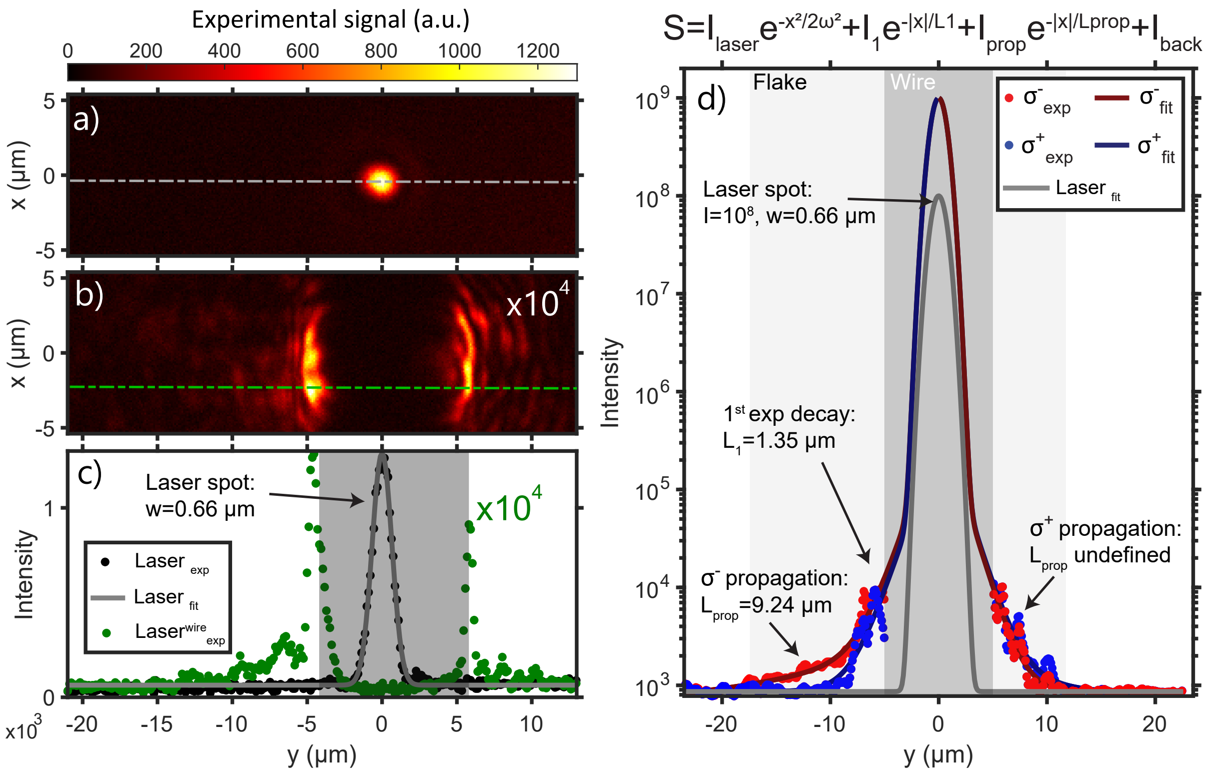

FIG. S12. Propagation length estimation from cross-sections of the zigzag structure propagation measurements. a-c) Estimation of the laser waist and the signal reduction when placing a wire as a spatial filter (see Fig. S9). a) Laser spot signal on the structure away from the interface with ND filters reducing the signal by 10^4. b) Laser signal on the same region with the wire as a spatial filter, and without the ND filters. Consequently, the signal collected at the border of the wire is 10^4 weaker than the laser spot. c) Cross sections of the two previous measurements in a-b) indicated by the dashed gray and green lines. The laser spot was fitted with a Gaussian line $S = I_{Laser}e^{-x^2/2\omega^2}$, giving a laser spot waist of 0.66 μm. d) Cross-sections taken from the measurements shown in Fig. S10a-b) in a range of y in which the interface propagation signals is highest. The cross-section of the counter-clockwise polarization measurements (σ^-, Fig. S10 a)) is plotted as red dots, and the clockwise polarization (σ^+, Fig. S10b)) as blue dots. The cross-sections were fitted the same way as for the simulation (see Fig. S11) with $S = I_{Laser}e^{-x^2/2\omega^2} + I_1e^{-|x|/L_1} + I_{prop}e^{-|x|/L_{prop}} + I_{back}$. The laser waist of 0.66 μm obtained in c), and a laser intensity of 10^8 ($10^4 \times max(signal_{exp})$) were used as fixed parameters. In the case of the σ^+ (blue) signal for positive x, the poor contrast between the propagating mode signal and laser spot signal prevents from obtaining a good fit and a good value of propagation length. However a very good match is obtained between the experimental σ^- (red) signal for negative x and the model, which gives a propagation length of 9.24 μm.

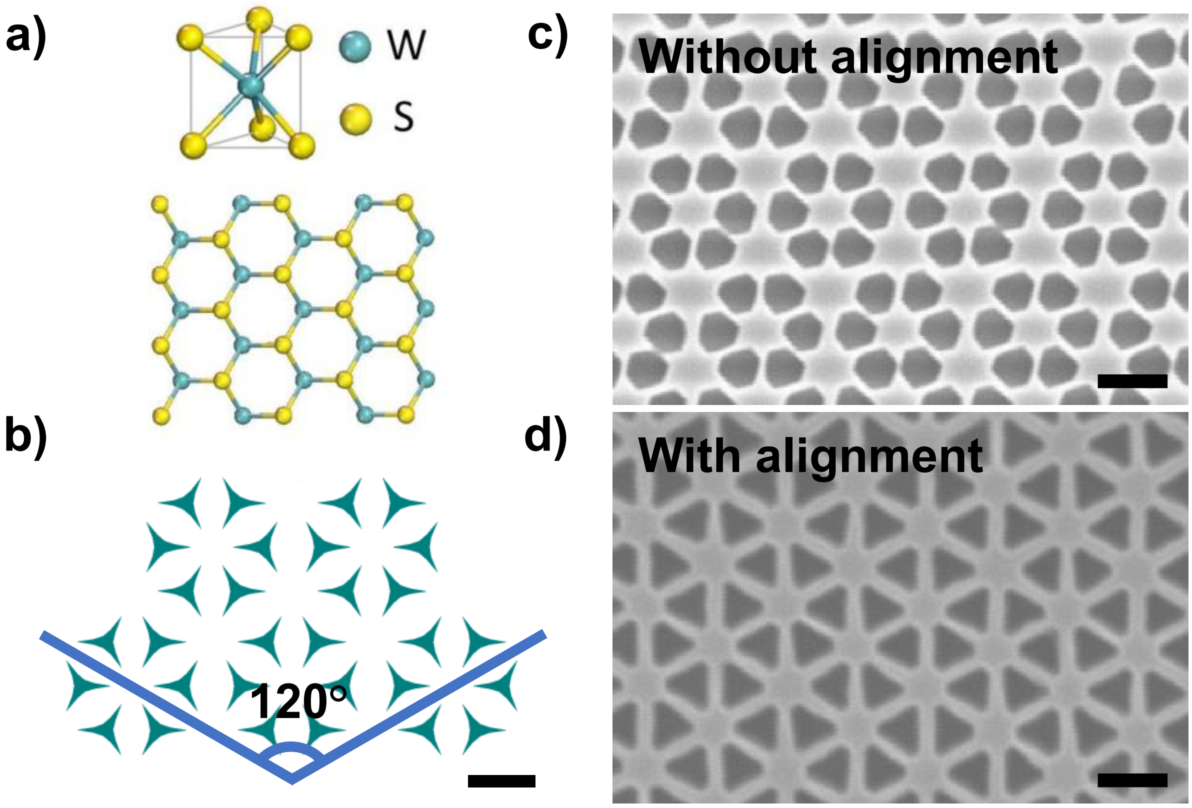

FIG. S13. a) Hexagonal crystal structure of WS_2. b) Dose pattern with shrunken lattice elements using an optimized pointed triangle design. The alignment of the pattern to the crystal axis of the WS_2 flake is shown, with the pattern aligned to a visible corner (blue lines) on the edge of the flake. SEM images of a c) fabricated structure without WS_2 crystal alignment and a simple straight-sided dose pattern for triangles and d) optimized structure using flake alignment and the optimized design. The optimized fabrication process as in d) was used for the structures in the main article. Nominal triangle side length s = 150 nm, a = 478 nm, scale bars 200 nm.

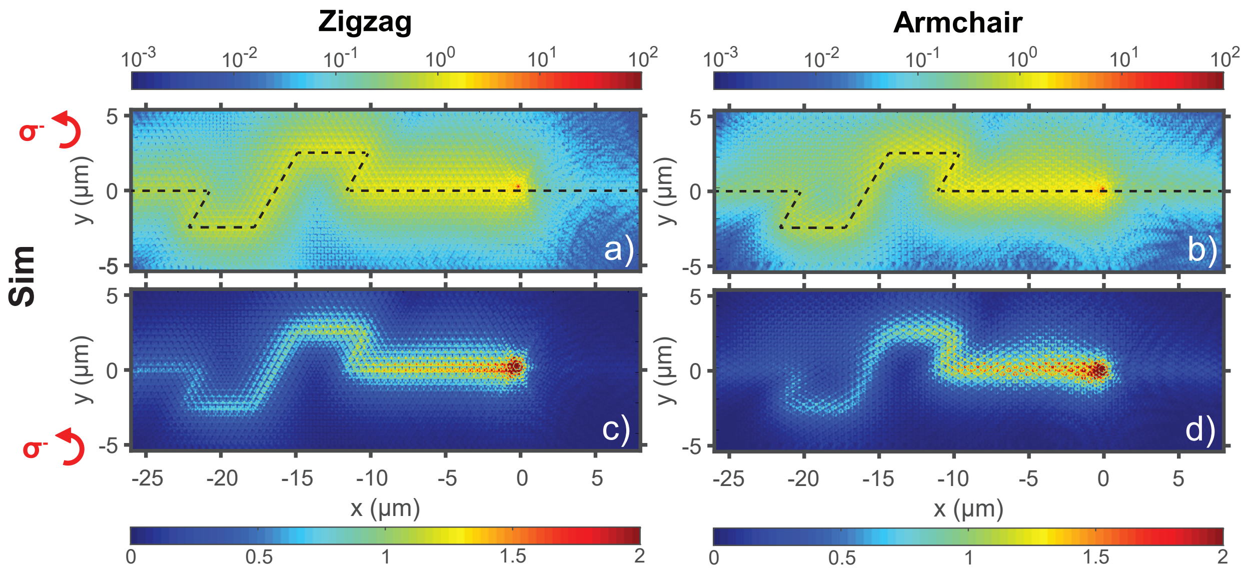

FIG. S14. Simulated propagation with the integrated electric field intensity at the center plane of the WS_2 flake over 1000 fs following excitation with counterclockwise polarization using a dipole pair at x = 0. Meandering interfaces in the a) zigzag and b) armchair directions are used with $|E|^2$ plotted using a logarithmic scale. The properties of the two samples are the same as in the main article, detailed in Fig. 2. The interfaces including the bends are shown with dashed lines. c,d) The same results using a linear scale for intensity. The route from $x = -5$ μm to -25 μm, a 30 μm distance along the interface, has an overall propagation length of 32 ± 5 μm for the zigzag and 22 ± 5 μm for the armchair structures.

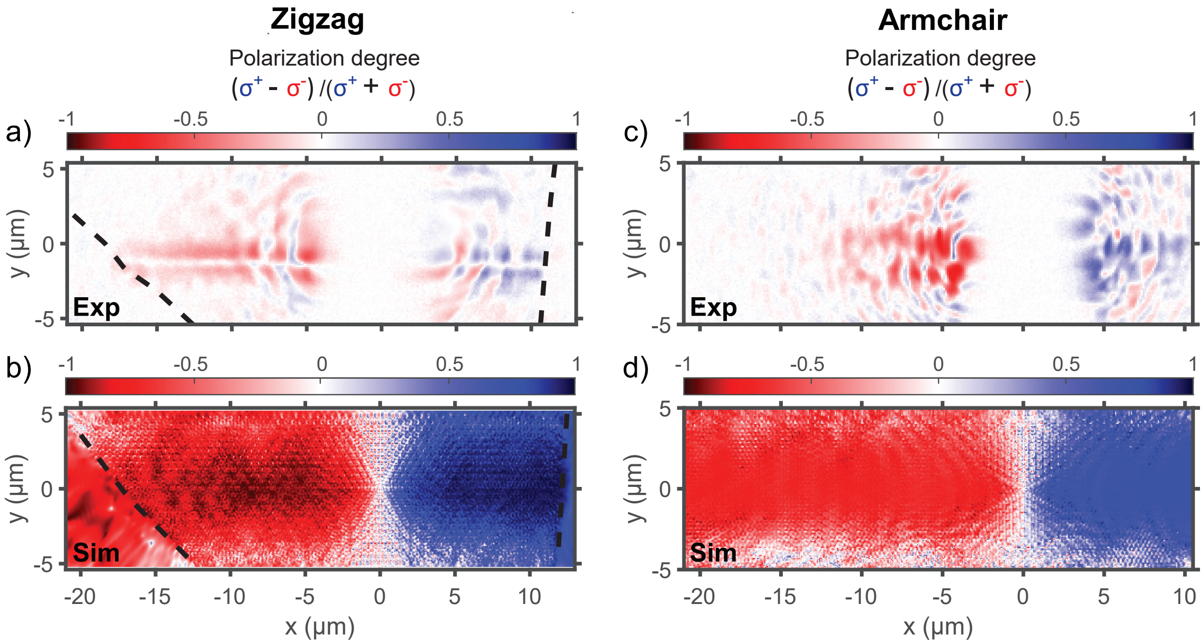

FIG. S15. Degree of circular polarization $[(\sigma^+ - \sigma^-)/(\sigma^+ + \sigma^-)]$ along the domain boundary in the spin-Hall WS_2 structure. Experimental degree of circular polarization for TE (E-field in plane) polarization for a) zigzag and c) armchair structures. b,d) Corresponding degree of circular polarization of the simulated electric field in the zigzag and armchair structures. The black dashed lines in a) and b) indicate the border of the flake of the zigzag structure (see Fig. 2a in the article). The white area in the middle of the experimental signals correspond to the spatial filtering used to suppress the direct intense reflection from the input laser.

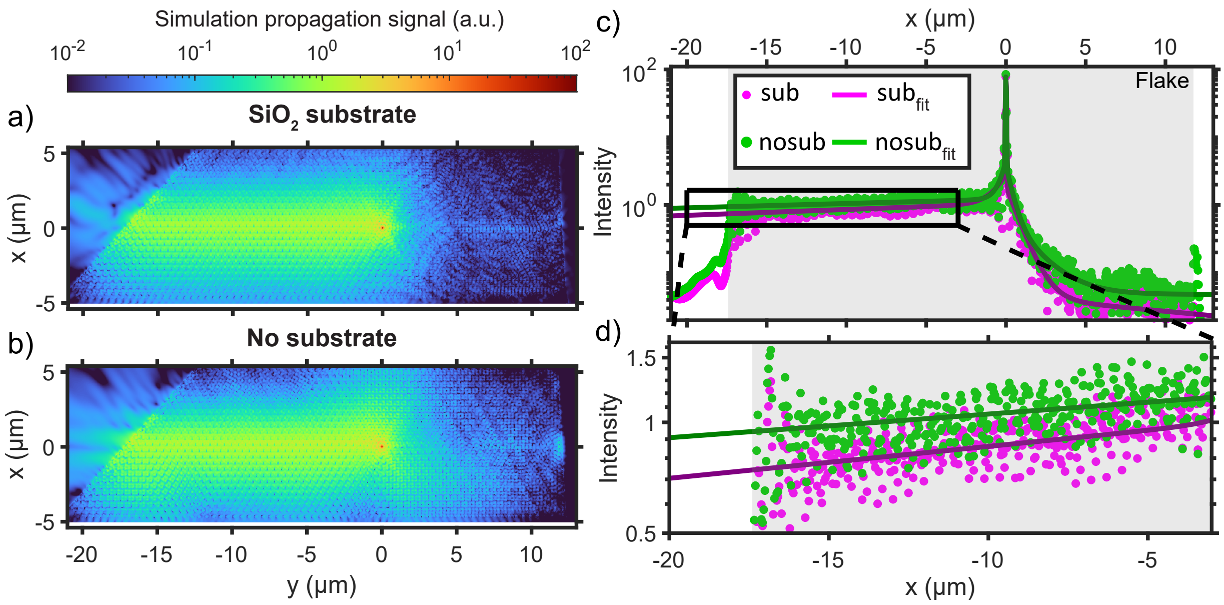

FIG. S16. Effect of the substrate on the propagation length of the simulated zigzag structure. a-b), FDTD simulated propagation (integrated E field at the center plane of the WS_2 layer) for clockwise polarization of the zigzag structure on a SiO_2 substrate (a), and suspended zigzag structure (b). c) Propagation analysis of the zigzag structures in a) and b) using the same method as in Figure S11. d) Zoom-in on the propagation region of c). The exponential decay fits give a propagation length of 50.12 μm for the zigzag structure on a SiO_2 substrate in a), and 64.30 μm for the suspended structure in b). The propagation length is slightly reduced due to the presence of a substrate as the refractive index contrast between the guided mode slab and the media surrounding it reduces the confinement of the mode.

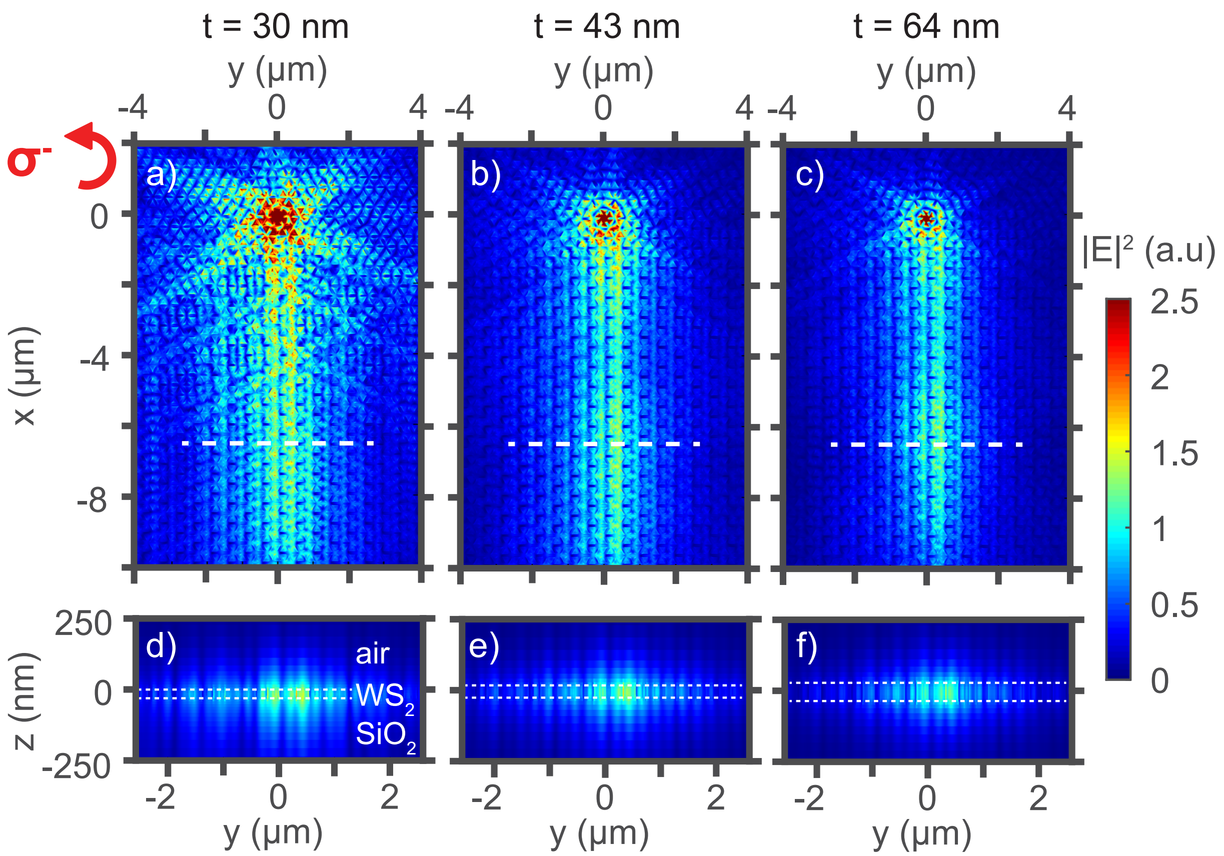

FIG. S17. Simulated propagation with the integrated electric field intensity over 200 fs following the excitation with counterclockwise polarization using a dipole pair at x = 0. We use a zigzag interface with a = 560 nm, s = 182.5 nm, c = 0, $R_s = 0.95R_0$ on left side, $R_e = 1.05R_0$ on right. WS_2 thickness and monitor wavelength changed to: a) t = 30 nm, λ = 807 nm, b) t = 43 nm, λ = 897 nm, c) t = 64 nm, λ = 978 nm. d-f) Corresponding $|E|^2$ in cross section at locations marked at x = -5 μm in a-c), layer interfaces marked with thin dashed lines. The wavelength λ is chosen for the best selectivity in propagation for the optimal interface mode. As seen in the plots in panels a)-c), lower thicknesses correspond to worse selectivity in propagation along the interface.

REFERENCES

[1]M. Li, I. Sinev, F. Benimetskiy, T. Ivanova, E. Khestanova, S. Kiriushechkina, A. Vakulenko, S. Guddala, M. Skolnick, V. M. Menon, D. Krizhanovskii, A. Alù, A. Samusev, and A. B. Khanikaev, "Experimental observation of topological Z2 exciton-polaritons in transition metal dichalcogenide monolayers," Nat. Commun. **12**, 4425 (2021).
